# KDM4B: A Nail for Every Hammer?

**DOI:** 10.3390/genes10020134

**Published:** 2019-02-12

**Authors:** Cailin Wilson, Adam J. Krieg

**Affiliations:** 1 Department of Pathology, University of Kansas Medical Center, Kansas City, KS 66160, USA; cailin.wilson@gmail.com; 2 Department of Obstetrics and Gynecology, Oregon Health and Science University, Portland, OR 97239, USA; 3 Division of Reproductive and Developmental Sciences, Oregon National Primate Research Center, Beaverton, OR 97006, USA

**Keywords:** histone demethylases, KDM4B, hypoxia, cancer, development, ovary

## Abstract

Epigenetic changes are well-established contributors to cancer progression and normal developmental processes. The reversible modification of histones plays a central role in regulating the nuclear processes of gene transcription, DNA replication, and DNA repair. The KDM4 family of Jumonj domain histone demethylases specifically target di- and tri-methylated lysine 9 on histone H3 (H3K9me3), removing a modification central to defining heterochromatin and gene repression. KDM4 enzymes are generally over-expressed in cancers, making them compelling targets for study and therapeutic inhibition. One of these family members, KDM4B, is especially interesting due to its regulation by multiple cellular stimuli, including DNA damage, steroid hormones, and hypoxia. In this review, we discuss what is known about the regulation of KDM4B in response to the cellular environment, and how this context-dependent expression may be translated into specific biological consequences in cancer and reproductive biology.

## 1. Introduction

Once thought to be too chemically stable to be enzymatically reversed, methyllysine residues are now recognized as dynamic components of the histone code [1,2]. The characterization of LSD1 (KDM1A) as the first histone demethylase in 2004 was quickly followed by the discovery that the Jumonji-domain family of genes constitutes a diverse group of histone demethylases capable of reversing many of the known methyllysine residues in histones [3,4,5,6]. The founding gene *Jumonji*, named for the cruciform neural plate defect caused by genetic deletion in mice, contains a domain similar to the transcriptional co-repressor RBP2 (now known as JARID1A) [7,8]. By sequence comparison, this C-terminal portion of the Jumonji domain (JmjC) was shown to be a member of the large and diverse superfamily of non-heme iron-dependent dioxygenases [8,9]. Currently, there are 30 proteins classified by their JmjC-domain in humans [9]. Of these, 22 act as lysine specific histone demethylases (KDMs) [10].

Similar to other dioxygenases, the basic protein structure of the JmjC domain is a β sheet “jelly roll” with iron II (Fe^2+^) coordinated in the central active site [8]. The majority of JmjC proteins use 2-oxoglutarate (2-OG, or α-ketoglutarate) and molecular oxygen (O_2_) as substrates to hydroxylate methyllysines in the N-terminal tails of histones, with CO_2_ and succinate as products. The resulting hydroxy-methyllysine is chemically unstable and rapidly degrades to formaldehyde and a lysine with a reduced level of methylation. The JmjC domain is frequently accompanied by an N-terminal domain (JmjN) that may contribute to substrate specificity and regulatory function [11,12]. When grouped by similarity of the JmjC domains and flanking sequences, there are six subfamilies of JmjC- KDMs (KDM2-KDM7), multiple histone demethylases not yet classified into distinct families (MINA53, NO66, KDM8), with the remaining members of the family hydroxylating non-histone substrates (JMJD6 and HIF1AN) or with uncharacterized catalytic activity (HSBAP1, JMJD7, JMJD8). The founding member of the JmjC family, now known as JARID2, lacks the key histidine and aspartate residues necessary to coordinate Fe^2+^ yet functions as a co-repressor in the polycomb silencing complex [13,14,15]. For a more comprehensive survey of the various JmjC-KDMs, we direct the reader to several excellent reviews [1,9,10]. For the purpose of this review, we will focus on KDM4B, a member of the KDM4/JMJD2B histone demethylase family notable for its diverse expression patterns in several tissues and pathological states. 

## 2. KDMB is a Histone Demethylase that Reverses Repressive Histone Modifications

**Figure 1:**
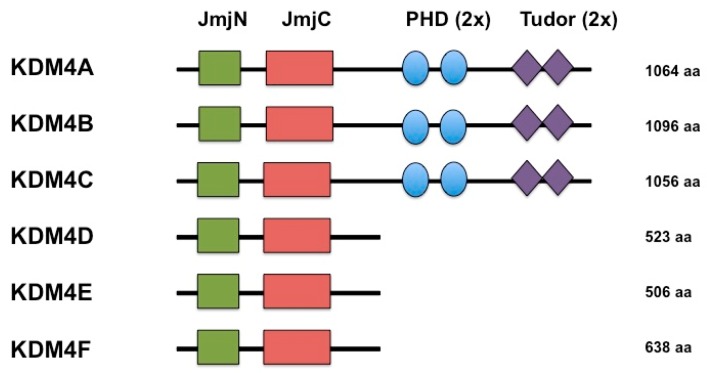
Diagram of the comparative domain structures of the KDM4 histone demethylases (adapted from Katoh and Katoh, 2004).

KDM4B is a member of the KDM4/JMJD2 family of histone demethylases. The KDM4 family contains four genes (*KDM4A*-*KDM4D*) and two pseudogenes (*KDM4E* and *KDM4F*) (Figure 1). KDM4A, KDM4B, and KDM4C are very similar in overall protein structure, each containing a JmjN domain, JmjC domain, two Plant Homeodomains (PHD), and two Tudor domains. KDM4D and KDM4E are smaller and lack the C-terminal PHD and Tudor domains [5,16,17]. The JmjC domain facilitates catalytic activity for these proteins while the JmjN domain may serve as a dimerization domain and provide structural integrity [5,16,17,18]. *KDM4B* localizes to the human chromosome 19p13.3 [16]. The full-length KDM4B protein is 1096 amino acids in length with catalytic activity against the histone residues H3K9me3, H3K9me2, H3K36me3, H3K36me2, H4K20me2, and H1.4K26me3, although H3K9me2/3 is the preferred substrate [16,19,20]. Like the rest of the KDM4 family, KDM4B can demethylate tri- and di-methyllysine to the monomethyl state [21,22]. Although the majority of epigenetics literature associates di- and tri- methylation of H3K9 with heterochromatin and gene repression [23,24,25,26,27,28], there is also evidence that H3K9 methylation located within gene bodies serves to facilitate gene expression [29,30]. Furthermore, the degree of H3K9 methylation correlates with nuclear position of chromatin, with H3K9me1 associated with open transcribed chromatin, H3K9me2 associated with nuclear lamina, and H3K9me3 corresponding to condensed chromatin [31,32,33]. KDM4B induction in specific contexts would then be predicted to remodel nuclear localization and transcriptional activity of specific gene regions by demethylating H3K9me2/3.

In general, loss of one KDM4 family member is not sufficient to distort total histone methylation, suggesting that each family member regulates a specific set of genes in a specific cell type or condition [23,34,35,36]. ChIP-Seq mapping of Kdm4b and Kdm4c in murine embryonic stem cells showed that regions bound by Kdm4b alone or in combination with Kdm4c were largely associated with transcriptionally active genes, while sites bound by Kdm4c alone were associated with repressed regions [34]. In contrast to other studies [37], loss of Kdm4b or Kdm4c was not associated with promoter-specific changes in H3K9me3 [34]. Further research is required to determine if genes induced by loss of KDM4B are the result of direct repression or indirect loss of a repressor positively regulated by KDM4B [34,36,38]. Non-histone substrates have also been identified for KDM4A-C [39]. All three enzymes demethylate the transcriptional repressors WIZ (ZNF803), CSB (ERCC6), CDYL1, and G9a (EHMT2) with higher specific activities than for methylated H3K9me3 peptides [39]. In the case of KDM4B, the majority of its function is associated with the activation or maintenance of gene expression, implying that its demethylase activity serves primarily to reverse histone marks that repress expression of KDM4B target genes [40].

A great deal of what is known regarding the other protein domains in KDM4B has been inferred from KDM4A function: The Tudor domains of KDM4A recognize H3K4me3/me2 and H4K20me3/me2 [41]. KDM4B tends to have lower affinity for H3K4me3 and other marks in vitro [42], but has been shown to be recruited to H4K20me3/2 at sites of DNA damage [43]. More recently, the Tudor domains of KDM4B have been shown to bind to H3K23me2/3, potentially recruiting KDM4B to meiotic heterochromatin in order to more efficiently demethylate H3K36me3 [42]. This differential recognition of specific histone marks by the Tudor domains of the KDM4 family may enable preferential recruitment to chromatin domains, targeting histone demethylation events to specific regions, and thereby influencing biological phenomena. The complexity of the KDM4B molecule, its variable catalytic and binding activities, and diverse expression mechanisms make it a flexible regulator of chromatin structure and biological processes.

## 3. KDM4B is Regulated by Multiple Cellular Stimuli

**Figure 2:**
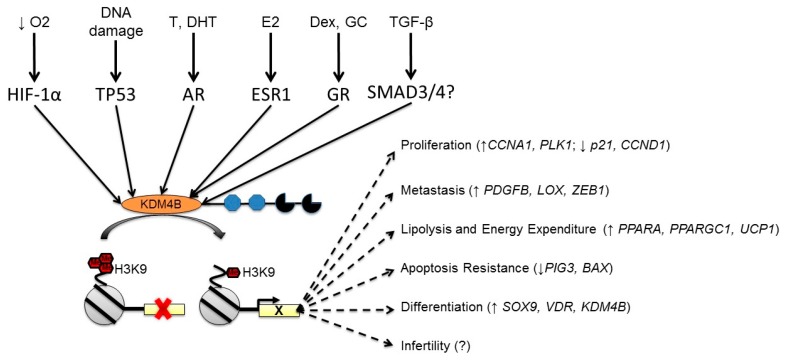
KDM4B integrates multiple cellular signals to affect biological processes. KDM4B is induced by multiple different extracellular stimuli. The majority of studies regarding its function describe it as an activator that removes repressive H3K9me3 and H3K9me2 at or near regulated promoters in order to facilitate expression of the indicated pathways. Representative target genes (if known) are shown in each pathway.

A key aspect of mediating the function of KDM4B (or any protein) is regulating the overall expression levels of said protein. While KDM4A is one of the best-studied JmjC-KDMs at the level of catalytic mechanism and structural analysis, KDM4B has tended to receive more attention at the level of transcriptional regulation and downstream function [40]. In particular, KDM4B is regulated by hypoxia, steroid hormone receptors, DNA damage, and multiple other cellular stimuli. These expression phenomena may work in isolation or in concert to tune the cellular response (Figure 2). 

### 3.1. Regulation of KDM4B by Hypoxia

Shortly after the discovery of JmjC-KDMs, multiple reports demonstrated the hypoxic regulation of several histone demethylases [44,45,46]. Given the strong link between tumor hypoxia and poor patient outcome, these discoveries made compelling links between the cellular microenvironment, epigenetic regulation, and tumor progression [47]. Hypoxia drives tumorigenic changes within cells and it also selects for cells equipped to survive fluctuations in oxygen availability [48]. Hypoxia occurs in cells approximately 100–200 µm away from a functional blood supply and at sites of heterogeneous tumor blood flow [49,50]. Tumor hypoxia is associated with therapeutic resistance driven by increased metastatic behavior, increased angiogenesis, reduced proliferation, increased glycolysis, and resistance to apoptosis [51,52,53]. All of these pathways contribute to therapeutic resistance, and many genes that constitute these pathways are directly regulated at the transcriptional level by Hypoxia Inducible Factors (HIFs) [53].

The HIFs are heterodimeric basic helix-loop-helix transcription factors that directly regulate the expression of hundreds of genes required for adaptation to hypoxia [54,55,56]. The primary mode of regulation for these proteins is oxygen-dependent degradation of the α subunit (HIF-1α and HIF-2α) mediated by prolylhydroxylases (PHDs) [57,58]. When sufficient oxygen and 2-OG is available, PHDs hydroxylate prolines 402 and 564 of HIF-1α and prolines 405 and 531 of HIF-2α [59,60,61]. The conformation shift imposed by the 4-hydroxyprolines creates a recognition motif for the Von Hippel Lindau tumor suppressor (VHL), an E3 ubiquitin ligase that promotes degradation of HIFs through the proteasome [62,63,64]. Reduction of O_2_ or 2-OG in the hypoxic microenvironment permits the rapid accumulation of HIF-1α and HIF-2α, their subsequent dimerization with the Arylhydrocarbon nuclear translocator (ARNT or HIF-1β), and binding to Hypoxia-responsive elements (HREs) in the promoters of hypoxia-inducible genes [65,66]. KDM4B is directly induced by HIF-1α from an HRE located approximately 500 bp upstream of the transcriptional start site [44,45,46,67]. The hypoxic induction of KDM4B in multiple cell types makes it a particularly compelling target for studying epigenetic regulatory mechanisms in various cancers.

The regulation of KDM4B by hypoxia raises interesting questions regarding its downstream effects on gene expression. Because all JmjC enzymes require oxygen for their catalytic activity, it is tempting to hypothesize that hypoxic induction of KDM4B serves to compensate for reduced substrate availability. However, in one of the earliest descriptions of the hypoxic regulation of KDM3A and KDM4B, Beyer et al. demonstrated that while both KDM3A and KDM4B were functional histone demethylases in 1% O_2_, only KDM3A was functional in 0.2% O_2_ [44]. More recently, KDM4A was shown to have an apparent Km (O_2_) of 176 µM (roughly equivalent to 18% O_2_ at 37 °C), indicating that catalytic activity of KDM4 enzymes is highly sensitive to changes in cellular oxygen [68]. At 1% O_2_, KDM4A was 40% less effective as a histone demethylase than at 21%, and had virtually no activity at 0.1% [68]. KDM4 enzymes are less sensitive to O_2_ changes compared to the prolyl hydroxylases, which have Km (O_2_) ranging from 230–450 uM, depending on the study [69,70]. In this respect, the KDM4 family may be more similar to FIH (Factor Inhibiting HIF), another JmjC domain protein with glutamine hydroxylase activity (Km (O_2_) of 90–110 mM) [69,70]. The histone demethylase activity of KDM4B will likely attenuate significantly within a hypoxic microenvironment. Nevertheless, siRNA-mediated knockdown of KDM4B in MCF7 and SKOV3ip.1 cells demonstrate that loss of KDM4B in hypoxic conditions has profound effects on downstream gene expression, and that many of the genes regulated under standard oxygen are distinctly different from those regulated by hypoxia [35,36]. Furthermore, while the genes positively regulated by KDM4B under hypoxia included a subset of HIF targets, glycolytic genes were not generally altered, implying that KDM4B is not a general co-regulator for HIFs [35]. When interpreted in light of recent reports that Kdm4b positively regulates lipolysis and mitochondrial energy expenditure, it appears that KDM4B contributes more to aerobic metabolism than anaerobic metabolism [71,72].

Combined with evidence of reduced catalytic activity in hypoxia [44], it remains to be determined if induction of KDM4B regulates hypoxic gene expression solely through histone demethylation, or if it also can play a structural role in nucleating larger transcriptional complexes, as in the case of JARID2 [13,14]. The association of Kdm4b with Myc- and Nanog-regulated promoters in the apparent absence of H3K9me3 changes supports this possibility [34]. Additionally, hypoxic induction of KDM4B might load a regulatory region with histone demethylases under hypoxic conditions, leaving it “poised” to demethylate histones once oxygen is restored. Because hypoxic induction of KDM4B is observed in many cell types, there remain ample opportunities to investigate the specific mechanisms of gene expression and DNA repair regulated by KDM4B in hypoxia.

### 3.2. Regulation of KDM4B by Nuclear Hormone Receptors

In one of the earliest characterizations of KDM4B function, Yang et al. convincingly demonstrated that KDM4B was induced by both HIF-1α and estrogen receptor α (ESR1) in MCF7 breast cancer cells [36]. Experiments using human cancer cells and knockout mouse models demonstrated a role for KDM4B as an essential factor for estrogen-dependent gene expression and for growth and differentiation of the mammary epithelium [73,74]. This establishes KDM4B as a significant contributor to estrogen-dependent gene expression; in response to estrogen, KDM4B is induced, and the resulting protein binds the promoters of some estrogen regulated genes, regulating both itself, *ESR1*, *FOXA1*, and genes that regulate proliferation of breast cancer [75]. Since many of the estrogen-induced genes that regulate breast cancer proliferation and progression are also regulated by hypoxia (*Cyclin D1*, *WISP2*, etc.), KDM4B serves to integrate two known drivers of breast cancer progression [40]. Effectively targeting KDM4B in ER+ breast cancer patients could prevent Tamoxifen resistance by preventing re-expression through hypoxic signaling. Although less well studied, there are indications that glucocorticoid receptors enhance the ESR1-dependent induction of KDM4B in breast cancer [76]. In this case, breast cancer cells have a more differentiated phenotype, inducing expression of *KDM4B* and *VDR* while preventing expression of Wnt-signaling molecules [76].

KDM4B expression is also directly induced by androgens via the androgen receptor to promote a more aggressive prostate cancer phenotype [77,78]. In prostate cancer cells, KDM4B expression, which correlates with the severity of tumor types, can cooperate with AR to induce the AR response [77]. KDM4B also stabilizes AR through inhibiting its ubiquitination and degradation [77]. Knockdown of KDM4B results in decreased AR expression [77]. The progression of prostate cancer from androgen-dependent to androgen-independent is one characteristic of this disease’s lethal progression [79]. These observations suggest that KDM4B may be a useful therapeutic target in prostate cancer for both androgen-dependent and androgen-independent tumor types [80]. Inhibition of KMD4B in combination with other chemotherapeutic drugs may improve current treatment options for prostate cancer.

### 3.3. Regulation of KDM4B by DNA Damage

In addition to being induced by pro-tumorigenic regulatory mechanisms, KDM4B has also been shown to be induced by the tumor suppressor p53 [38,81]. When activated by DNA damage or the chemical inducer nutlin, p53 directly binds the p53RE located approximately 500 bp upstream of the KDM4B transcriptional start site [38]. This mode of regulation may be specific to humans and other non-human primates: Murine cells do not induce Kdm4b following DNA damage, and expression is not altered in p53 knock-out MEFs [38]. This is in contrast to the hypoxic induction of KDM4B in human and murine cells [44,45,67], as well as the estrogen-dependent induction of KDM4B in both breast cancer and murine mammary epithelium [36,74]. The functional consequences of this p53-dependent regulation remain to be completely determined, but in three independent manuscripts, loss of KDM4B increases the baseline expression of the p21 cell cycle inhibitor (*CDKN1A*), *PIG3*, and *PUMA* [38,81,82]. In a more comprehensive analysis, Castellini et al. demonstrated that KDM4B may act as a negative feedback regulator of p53 target gene expression, and that loss of KDM4B more effectively inhibited tumor growth in parental HCT116 cells compared to matched p53-KO cells [38]. Loss of KDM4B may also activate p53 in gastric cancer cells, resulting in increased expression of p21 and reduced proliferation [82].

**Figure 3:**
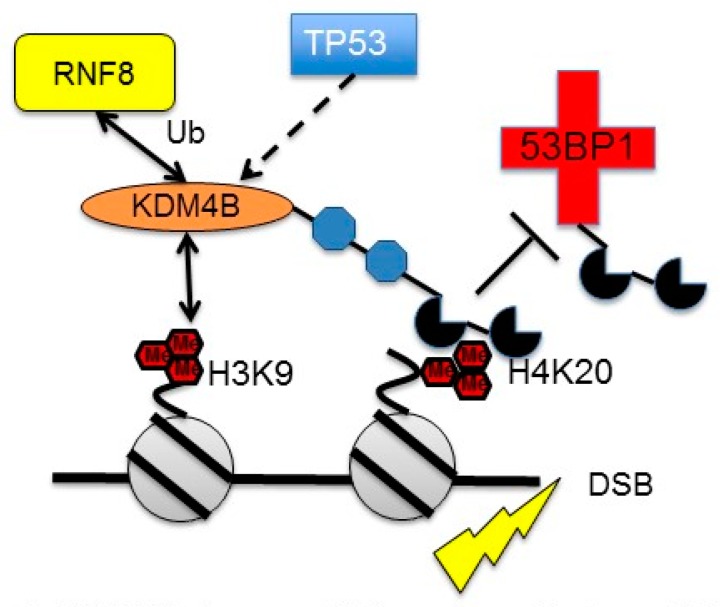
KDM4B influences DNA repair mechanisms. KDM4B is directly induced by p53. The Tandem Tudor domains target KDM4B to H4K20me3/2 at sites of DNA damage. This recruitment blocks the binding of 53BP1 to the same marks until RNF8 removes KDM4B through ubiquitylation. KDM4B can also be recruited to heterochromatin in response to DNA damage, removing H3K9me3/2 to facilitate DNA repair. (Adapted from Young et al., 2013, Mallette et al., 2012 and Zheng et al., 2013).

Induction of KDM4B by DNA damage may facilitate DNA repair by decreasing levels of H3K9me3 in pericentric chromatin [81]. Simultaneous downregulation of *SUV39H1* by p53 further reinforces the demethylation process [81]. In a separate study, KDM4A and KDM4B were both shown to localize to H4K20me2 in response to DNA damage [43]. This localization slowed recruitment of 53BP1 to sites of damage and was reversed by ubiquitylation of KDM4A/B by RNF8 (Figure 3). While Mallete et al. concluded that slowing 53BP1 recruitment would increase DNA damage [43], one could also speculate that blocking 53BP1 recruitment could slow non-homologous end-joining (NHEJ). In non-transformed cells, this could increase the efficiency of homologous recombination and other higher fidelity mechanisms, resulting in reduced genomic dysregulation [83]. A role for KDM4B has been proposed in the transient amplification of chromosome 1p32.3 during replication, a possible mechanism of generating DNA damage [84]. KDM4B was shown to directly interact with replication factors and components of the DNA polymerase complex directing selective re-replication of 1p32.3 [84]. This mechanism appears to be similar to the site-specific amplification of chromosome 1 (1q12h, 1q21.2, 1q21.3) mediated by KDM4A in hypoxia and during DNA replication [84,85,86]. Overexpression of KDM4B has also been shown to induce DNA damage by activating LINE-1 elements [87]. How these effects of KDM4B expression integrate with the induction of KDM4B by other stressors remains to be determined.

The regulation of KDM4B by p53 has primarily been observed in transformed cells expressing wild-type p53 (i.e. HCT116 colon carcinoma, U2OS osteosarcoma cells, and gastric cancer cells) [38,81,82]. It remains to be determined if non-transformed cells display this same mechanism of regulation. Nevertheless, several testable hypotheses can be proposed: Does hypoxic and hormone-dependent regulation of KDM4B attenuate the cell cycle arrest and apoptosis functions of p53 sufficiently to permit transformation and sustain tumorigenesis? In non-transformed cells, does suppression of KDM4B drive cell cycle arrest and senescence sufficiently to prevent tumorigenesis? Could KDM4B induction by p53 represent another facet of DNA repair regulated by p53? How does DNA damage induced by over-expression of KDM4B in some models fit into its regulation by p53? The absence of p53-dependent regulation in murine cells makes it more difficult to construct a genetically tractable model to test these hypotheses. Continued interest in KDM4B will also doubtless identify other modes of regulation, such as induction by CREB and TGF-β [88,89]. Future experiments are required to reconcile these pieces of data in a manner reflective of the conditional nature of KDM4B expression and the effect of cell background.

## 4. Integrating KDM4B Expression Phenomena with Biological Processes

### 4.1. KDM4B and Stem Cell Biology

Epigenetic modifiers are important contributors to developmental processes [90], and KDM4B is no exception. KDM4B is expressed in undifferentiated and differentiated ESCs [91]. ESCs are indefinitely self-renewing and differentiating cell populations, with identities maintained or differentiation controlled by activation of specific genes through transcription factors or chromatin regulators [34,90,92]. Kdm4b participates in embryonic stem cell self-renewal and induced pluripotent stem cell generation in mouse models by associating with Nanog-regulated promoters [34]. Kdm4b and Kdm4c work synergistically to mediate gene expression during differentiation of mouse ESCs [34]. H3K9me3 marks in heterochromatin are very stable and resist reprogramming. Thus, reduction of H3K9me3 levels by Kdm4b improved in vitro development of cloned embryos [93]. Ectopic introduction of KDM4D to somatic cell nuclear transfer experiments has resulted in more efficient cloning of macaque embryos [94]. There is evidence that Kdm4b and Km4d may have overlapping and compensatory roles in spermatogenesis [95]. Future investigations into KDM4B and KDMD function during gametogenesis should determine the specific mechanisms regulated by each enzyme to remodel chromatin during gametogenesis and embryogenesis.

### 4.2. KDM4B and Mesenchymal Tissues

KDM4B regulates mesenchymal stem cell (MSC) fate, identifying a possible control mechanism to exploit in directing MSC lineage [96,97]. MSCs are multipotent progenitor cells with multi-lineage differentiation potential [96]. KDM4B controls MSC differentiation and fate by removing H3K9me3 to activate *DLX* [96,97]. Loss of KDM4B reduced osteogenic differentiation and increased adipogenic differentiation [96,97]. BMP4 induces DLX5 and KDM4B in stem cells of the apical papillae to mediate osteo-dental differentiation [98]. Regulation of KDM4B by SMADS and DLX5 establishes a positive feedback mechanism to maintain expression of DLX5 [98]. This provides a promising therapeutic target for diseases such as osteoporosis, which are commonly associated with a loss of MSC commitment [96,97].

KDM4B participates in chondrogenesis and adipogenesis in MSCs [89,99]. TGF-β dependent KDM4B expression removes the repressive H3K9me3 mark from the *SOX9* promoter, inducing expression of one of the master regulators of chondrogenesis [89]. In adipogenesis, KDM4B binds C/EBPβ (CCAAT/enhancer-binding protein) to promote mitotic clonal expansion, an important process preadipocytes enter as they differentiate [99,100]. KDM4B binds C/EBPβ, demethylates H3K9me3 to regulate expression of cell cycle genes *CDC45I*, *MCM3*, and *CDC25C* and to drive mitotic clonal expansion [99]. Although there were some differences between the respective models, knocking out *Kdm4b* in adipose tissue generally resulted in increased obesity, with increased expression of adipogenesis genes (e.g., *Pparg2* and *aP2*) and reduced expression of genes in energy expenditure and lipolysis pathways (e.g., *Ppargca1, Ppara*, *Acox1*, and *Atgl*) [71,72]. Re-expression of Kdm4b in *Kdm4b*-KO adipocytes required a functional demethylase domain to restore energy expenditure and cellular oxygen consumption, demonstrating that removal of H3K9me was required for target gene expression [72]. If KDM4B also regulates adipose differentiation in humans, selectively increasing expression in adipose tissue could help treat obesity.

### 4.3. KDM4B in the Central Nervous System

In addition to stem cell maintenance and differentiation, KDM4B is involved in neurobiological processes. KDM4B is a critical component in development of the central nervous system (CNS) [101]. Neuron-specific KDM4B knockout mice displayed neurodevelopmental disorders including spinal malformations and hippocampal impairment [101]. The hippocampus exhibited hyperactive behavior, deficits in working memory, and spontaneous epileptic seizures [101]. This *Kdm4b* knockout model provides a novel system for neurodevelopmental disorder in vivo investigations [101]. 

### 4.4. KDM4B in Ear Development

In addition to CNS development, KDM4B regulates inner ear invagination and ear development [102]. KDM4B is expressed during early stages of chick inner ear formation and loss of expression results in defective otic placode invagination and morphological changes [102]. KDM4B regulates *DLX3*, a marker for inner ear, by demethylating H3K9me3 in its promoter region [102]. This study provides one of the first connections between a histone demethylase and ear development.

### 4.5. KDM4B in Reproductive Tissues

KDM4B is linked to spermatogenesis and mammary gland development [74,103]. Increased Kdm4b and decreased H3K9me3 were observed during pre-spermatogenesis [103]. This suggests a role for KDM4B in reorganization of constitutive heterochromatin during a period of a key step of gametogenesis [103]. Kdm4b binds to estrogen receptor to regulate mammary gland development [74]. A mammary-epithelium specific conditional *Kdm4b* knockout mouse results in defective mammary gland development, identifying KDM4B as an important epigenetic component [74]. 

In the course of studying KDM4B in ovarian cancer, Wilson and Qiu et al. conducted control studies to detect KDM4B in normal ovarian tissue [35]. While ovarian surface epithelium showed little to no expression of KDM4B, there was robust expression in primordial follicles [35]. In follow up studies using archival ovarian cross-sections and granulosa cells collected from in vitro fertilization experiments, KDM4B and KDM4A mRNA was negatively correlated to successful pregnancy in IVF patients [104]. This was true for both the cumulus granulosa cells (the cells immediately adjacent to the oocyte in the developing follicle), and the mural granulosa (the cells lining the outside periphery of the follicle). Since granulosa cells are the primary producers of estrogen in the developing follicle, high expression of *KDM4B* would seem to be expected [105]. However, the strongest expression of KDM4B protein appeared to be in the early stages of folliculogenesis (i.e. primordial, primary, and secondary follicles) not in the later, antral stages [104]. It is not clear if this expression indicates that small follicles exist in a state of physiological hypoxia, if elevated *KDM4A* and *KDM4B* represents release of immature oocytes and granulosa during IVF, or if expression represents regulation by other stimuli. 

*KDM4B* mRNA is also elevated in the decidualized endometrial stroma of women with Recurrent Pregnancy Loss, compared to idiopathic miscarriage patients [106]. It is not clear if uterine KDM4B expression during miscarriage is related to its role in regulating the progression of endometrial cancer [107]. The expression of *KDM4B* in the ovary and uterus provides fertile ground for understanding the intersection between steroid hormones, hypoxic signaling, and DNA damage pathways in the Reproductive Endocrinology and Infertility field, with significant potential to impact egg quality and embryo development.

### 4.6. KDM4B in Non-Mammalian Systems

In addition to mammalian systems, KDM4B has been implicated in regulating *Drosophila* development [108,109]. KDM4B is essential for mediating ecdysteroid hormone signaling during *Drosophila* larval development [108]. KDM4A and KDM4B demethylate H3K9me3 specifically at the promoter of genes responsive to ecdysone signaling, such as Broad Complex (*BR-C*), allowing increased expression [108]. Double knockout of *Drosophila KDM4A* and *KDM4B* results in developmental arrest [108]. Rescuing these double knockouts with expression of either KDM4A or KDM4B demonstrates that at least one of these proteins are essential for *Drosophila* development and survival [108].

## 5. KDM4B in Cancer

In addition to developmental processes, KDM4B has been shown to play a significant role in cancer progression. In general, when the activity and expression levels of histone demethylases are disrupted, neoplasms may form [110]. Many histone demethylases are deregulated during pathogenesis, either activating expression of oncogenes, repressing expression of tumor suppressors, altering DNA repair, disrupting chromosomal stability or interacting with key hormonal receptors that control proliferation [111]. The correlation between increased histone demethylase expression and tumor development has been established for many enzymes, such as LSD1 and KDM4C [112]. Optimizing inhibitors to specifically target JmjC containing KDMs have become a novel facet of therapy design [113]. Recent bodies of work have characterized the contributions of KDM4B to tumorigenesis in both solid and hematological tumor types [40,114]. In the following sections, we will attempt to synthesize what is known regarding the context-dependent expression of KDM4B as a mediator of tumor progression in multiple cancer types.

### 5.1. KDM4B in Breast Cancer

As discussed previously, KDM4B is induced by estrogen, making it important for progression of ER+ breast cancers [36,73,74,75]. The involvement of KDM4B in breast cancer has a clear contribution to the aggressive nature of the disease in ER+ subtypes [74]. KDM4B is highly expressed in estrogen receptor (ER)-positive, aggressive subtypes [36,45,74]. As discussed earlier in this review, the regulation of KDM4B by Glucocorticoid receptor (GR) and ER-α establishes a feed-forward mechanism to enhance estrogen-dependent signaling and proliferation in vitro and in vivo [76]. KDM4B is required for ERα dependent transcription, where loss of KDM4B decreases cell proliferation in vitro and tumor progression in vivo [36,73,76]. KDM4B may also coordinate with additional epigenetic regulators to regulate histone modifications. KDM4B can bind ER-α and MLL2 to coordinate demethylation and methylation of H3K4 and H3K9 [73]. KDM4B and ER-α can also bind SWI-SNF-B remodeling complex to mediate the expression of ER responsive genes, *MYB*, *MYC*, and *CCND1* [74]. The tumor suppressor miR-491–5p can bind *KDM4B* mRNA and slow ER-α+ breast cancer development [115]. FBXO22, a subunit of the SCF ubiquitylation complex, degrades KDM4B when complexed to ESR1 in the unliganded or Tamoxifen-bound states [116]. Inhibiting degradation of KDM4B by blocking FBXO22 increases transcription mediated by the hormone-independent AF1 domain of ESR1 [116]. Targeting KDM4B or its regulated pathways in ER+ breast cancer may enhance the actions of Selective Estrogen Receptor Modulators to prevent tumor growth and recurrence. Targeting KDM4B with the demethylase inhibitors Methylstat or ML-324 activates the Unfolded Protein Response in PTEN-negative Triple-negative Breast Cancer (TNBC), an aggressive and hormone-independent form of breast cancer, sensitizing these cells to PI3-Kinase inhibitors [117]. Thus, KDM4B may function as a therapeutic target for both ER+ and ER- breast cancers.

### 5.2. KDM4B in Prostate Cancer

In addition to cooperating with AR to promote androgen-dependent gene expression and tumor progression [77], KDM4B promotes prostate cancer development through an AR-independent mechanism. KDM4B activates the transcription of BMYB-target genes important for cell-cycle progression and tumorigenesis, such as Polo-like kinase 1 (*PLK1*) [80]. KDM4B expression interferes with HDAC inhibitor efficacy. When *KDM4B* was knocked out, the HDAC inhibitor trichostatin A (TSA) treatment enhanced induction of apoptosis [80]. These studies provide support for targeting KDM4B or its regulated pathways in prostate cancer.

### 5.3. KDM4B in Colorectal Cancer

KDM4B is highly expressed and is a known contributor to colorectal cancer (CRC) [118]. In addition to being regulated by HIF-1α in this model, KDM4B can act as an oncogene and induce tumorigenesis through a variety of mechanisms [44]. When induced by *PRL-3* a gene linked to CRC metastasis, KDM4B can promote CRC tumorigenesis by promoting proliferation, colony formation and migration of human colorectal cancer cells [118]. Silencing *KDM4B* induced DNA damage and triggered senescence, apoptosis, and cell cycle arrest through suppression of STAT3 signaling [119]. Recently, KDM4B was shown to be induced by CREB to suppress the DNA damage response mediated by STAT3 [88]. In addition, KDM4B suppression can drive CRC apoptosis through mitochondria-mediated and death receptor-mediated pathways [107]. KDM4B, in conjunction with TC4 also binds with β-catenin to regulate expression of the oncoproteins *JUN*, *MYC*, and *Cyclin D1* [120]. This significant role of KDM4B in regulating many aspects of CRC suggests that it could make a useful therapeutic target. 

### 5.4. KDM4B in Gastric Cancer

KDM4B is connected to gastric cancer carcinogenesis, lending itself as a possible novel biomarker or target for inhibition. Increased expression of KDM4B has been shown to drive gastric cancer proliferation, promote epithelial-mesenchymal transitions, and induce COX-2 dependent inflammation [121,122,123]. Similar to colorectal cancer, KDM4B functions in a complex with β-catenin to increase expression of genes involved in the epithelial-mesenchymal transition in a demethylase-dependent manner [124]. KDM4B can induce *Helicobacter pylori* infection and resulting gastric inflammation, one of the strongest risk factors for gastric cancer development [121]. β-catenin stimulates KDM4B expression that induces COX-2 expression in a histone demethylase-dependent manner [121]. KDM4B-dependent expression of miR-125b was recently shown to induce b-catenin nuclear translocation and promote gastric cancer metastasis [125]. These mechanisms establish KDM4B as a significant participant in gastric cancer.

### 5.5. KDM4B in Osteosarcoma

KDM4B has been connected to osteosarcoma tumorigenesis, a cancer type that effects young adults and is plagued with recurrence [80,126]. KDM4B expression has been shown to drive tumorigenesis and participate in the DNA damage response [80]. KDM4B can promote proliferation, migration, and invasion through induction of Fibroblast growth factor 2 (FGF2) [80]. Hsp90 has also been shown to stabilize KDM4B in osteosarcoma cell models, identifying Hsp90 inhibitors as a possible target for KDM4B driving tumor types [127]. KDM4B also participates in regulating the DNA damage response in osteosarcoma models [43,128]. After irradiation, KDM4B is recruited to DNA damage in a PARP1 dependent manner to remove H3K9me3 at sites of damage [128]. KDM4B activity can influence 53BP1 recruitment to DNA damage sites [43]. After recruitment to double strand breaks, KDM4B is marked by RNF8 and RNF168 and degraded in the proteasome [43]. Combined, these findings suggest that KDM4B is a novel risk factor or biomarker in osteosarcoma.

### 5.6. KDM4B in Hematological Tumors

The role of KDM4B activity in hematological cancers has been explored in acute myeloid leukemia (AML) and multiple myeloma (MM) [123,129]. AML with translocations of the mixed-lineage leukemia 1 (MLL1) gene are aggressive hematopoietic malignancies, developing resistance to chemotherapies [123]. Using KDM4A, KDM4B, and KDM4C triple knockout mice, KDM4 demethylation of H3K9me3 was shown to be required for MLL-AF9 translocated AML pathogenesis in vitro and in vivo [123]. A possible redundant role was identified for KDM4 family proteins in AML and non-transformed bone marrow [123]. When only KDM4C is knocked out in AML mouse models, leukemic cells survive [123]. *IL3RA* ectopic expression alleviated the need for KDM4 proteins for survival and was shown to be a critical downstream target for KDM4 proteins in AML [123]. These findings suggest that KDM4B and other KDM4 enzymes could be promising drug targets in AML.

Multiple Myeloma is a plasma cell neoplasm characterized by the accumulation of terminally differentiated monoclonal plasma cells in the bone marrow and is one of the most diagnosed hematological cancer types [129]. Evidence suggests epigenetics are involved in the gene expression changes driving MM progression [129]. Triptolide induces apoptosis in AML and was explored for its effects on histone methylation and antitumor effect on MM [129]. Triptolide treatment induced apoptosis, and decreased KDM4B and H3K9me2 expression in MM [129]. These findings are consistent with studies demonstrating that KDM4B may have a protective role in apoptosis, mediating the DNA damage response [129]. These observations suggest high KDM4B expression may drive MM progression, but this needs further exploration.

In contrast to it role in myeloma progression, KDM4B may play a tumor suppressive role in chronic lymphotic leukemia (CLL). KDM4B expression is decreased in CLL compared to control tissues, particularly in the more aggressive and resistant ZAP-70 positive CLL cells (130). KDM4A is increased in CLL, and KDM4C is unchanged but is lower in ZAP-70 positive cells [130]. This suggests that inhibiting KDM4B in hematological cancers may have conflicting outcomes, depending on the tumors being treated.

### 5.7. KDM4B in Gynecological Cancers

KDM4B has been poorly explored in gynecological cancers. Gynecological cancers, particularly high grade serous ovarian carcinomas, are plagued with late diagnoses and high metastatic tumor burden at the time of surgery [131]. The high tumor load at the time of primary surgery is also a contributing factor to the rapid rate of recurrence and incidence of chemotherapeutic resistance [132]. KDM4B has been shown to promote endometrial cancer progression by regulating androgen receptor, c-Myc, and p27^kip1^ [107]. KDM4B regulates genes associated with seeding of ovarian cancer cells to the peritoneal and omental tissues during metastasis (*PDGFB*, *LOX*, *LOXL2*, and *LCN2*) [35]. In this case, KDM4B was primarily studied as a contributing factor to high grade serous ovarian carcinoma (HGSOC) via hypoxic regulation of metastatic mechanisms. TP53 is mutated or lost in over 90% of HGSOC, removing one potential mode of regulation from consideration [133]. Similarly, although many HGSOC tumors express estrogen receptor, neither estrogen nor SERMS significantly influence patient outcomes [134,135]. Unfortunately, expression of *KDM4B* mRNA is not associated with either poor or improved prognosis in HGSOC [133]. This does not mean that KDM4B is not important for mediating tumorigenic mechanisms in ovarian cancer, but it does mean that expression does not correlate with prognosis during conventional platinum-Taxol therapies. More studies are required to determine if the mechanisms regulated by KDM4B in gynecological cancers overlap with pathways targeted by current and emerging chemotherapies.

### 5.8. KDM4B in Other Cancers

KDM4B activity is also linked to liver, lung, and bladder cancer development [118,119,136]. KDM4B shows increased expression correlating with tumor grade severity in Hepatocellular carcinoma (HCC) [136]. KDM4B is highly expressed in lung and bladder tissue compared to normal tissues [119]. Knockdown of KDM4B in lung and bladder cancer models showed decreased proliferation and decreased colony formation, through decreased expression of *CDK6* [119]. KDM4B has also been shown to regulate the N-Myc pathway in neuroblastoma [137]. KDM4B and N-Myc are highly expressed in neuroblastoma tumors and correlate with poor outcome [137]. Co-IP analysis showed binding between KDM4B and N-Myc [137]. When KDM4B is knocked down, there is decreased proliferation, differentiation and tumor growth in neuroblastoma models [137]. KDM4B expression and activity has also been explored in the progression of uveal melanoma [138]. In addition to aberrant expression of other histone modifiers, KDM4B was found to be down-regulated in uveal melanoma with monosomy 3 [138]. Monosomy 3 is one of the predictive markers of poor prognosis in this cancer type [139]. These findings implicate KDM4B as a general facilitator of tumor progression with diverse roles in mediating progression.

## 6. KDM4B as a Therapeutic Target (i.e., a Nail for Every Hammer)?

When analyzed in isolation, KDM4B regulates a broad suite of pathways that promote whatever pathological condition is the focus of study. This would seem to make it an excellent target for therapeutic development, particularly in the multiple cancers where KDM4B contributes to progression. Given the central importance of epigenetic regulation in tumor progression, inhibitors of KDM4B and many other JmjC-KDMs are being actively developed for clinical application [140,141,142,143,144,145,146,147]. We refer the reader to some comprehensive reviews describing the multiple types of KDM inhibitors currently in development, and the respective mechanisms of action [10,148]. Although there has been significant progress, the commonality of the reaction mechanisms between the various JmjC-KDM family members frequently results in inhibition of enzymes in other subfamilies [140]. In the case of the KDM4 family, where elevated expression seems to be a hallmark of many cancers, it may not be important to distinguish between KDM4A, KDM4B, KDM4C, and KDM4D. Specifically targeting KDM4B with shRNA increased KDM4D RNA and protein expression in ovarian cancer cells [35]. Targeting all KDM4 enzymes at once would prevent any compensatory expression, yielding maximum clinical efficacy. On the other hand, given the clear importance of KDM4B for basic cellular functions in normal tissues, particularly stem cell maintenance and proliferation, one might predict that inhibiting its activity could result in debilitating side effects comparable to those observed with existing anti-proliferative chemotherapies (e.g., gastrointestinal and renal dysfunction, neutropenia, and anemia).

A more promising avenue for targeting KDM4B may lie in mediating “synthetic lethality.” A subset of tumors harbor mutations in isocitrate dehydrogenase 1 and 2 (IDH1/2), resulting in the aberrant conversion of of α-ketoglutarate (2-oxoglutarate, 2-OG) to the the R enantiomer of 2-hydroxyglutarate (R-2-HG), an inhibitor of JmjC histone demethylases and TET DNA demethylases [149,150,151,152]. In recent studies, R-2HG is 3 times more effective at inhibiting KDM4B than S-2HG in vitro (Km of 150 µM vs. 450 µM) [153]. IDH mutant cells are more sensitive to PARP inhibitors (PARPis), increasing DNA damage and cell death [154]. Loss of KDM4A and KDM4B increases DNA damage in wild-type IDH backgrounds, while over expression of either reverses the effect of IDH mutation [154]. The Krebs cycle intermediates citrate, succinate and fumarate, can also inhibit JmjC-KDMs and promote defects in homologous recombination that increase sensitivity to PARPis (albeit with much higher Km than R-2HG) [153,155]. Although specifically restricting KDM4B inhibition to a tumor remains challenging, identifying conditions where KDM4B is inhibited by aberrant metabolite accumulation could more effectively direct the application of PARPis and other chemotherapeutics.

The old proverb “For a man with a hammer, everything looks like a nail” refers to the all too human tendency to address a new problem with the tools that we are most familiar. In the case of KDM4B, which is regulated by multiple stimuli, in multiple cell types, in multiple pathological conditions, it would seem to be an ideal “nail” for several therapeutic “hammers.” However, the contributions of KDM4B to such a broad spectrum of biological processes suggests that a great deal more research is required to convert its clear biological importance into the development of effective therapeutic strategies.
